# Longitudinal outcomes of severe post-stroke dysphagia: high mortality, partial recovery, and persistent dependence on alternative feeding

**DOI:** 10.1016/j.clinsp.2025.100825

**Published:** 2025-11-12

**Authors:** Gabriela Mourthé Félix, Fernanda Chiarion Sassi, Carina Escudero, Karoline Kussik de Almeida Leite, Ana Paula Ritto, Cirley Novais Valente Junior, Claudia Regina Furquim de Andrade

**Affiliations:** a Divisão de Fonoaudiologia do Instituto Central do Hospital das Clínicas, da Faculdade de Medicina da Universidade de São Paulo, São Paulo, SP, Brazil; b Departamento de Fisioterapia, Fonoaudiologia e Terapia Ocupacional da Faculdade de Medicina da Universidade de São Paulo, São Paulo, SP, Brazil

**Keywords:** Stroke, Dysphagia, Mortality, Enteral nutrition, Rehabilitation

## Abstract

**Objective:**

This study evaluated the progression of swallowing function and clinical outcomes in patients with severe dysphagia following Ischemic Stroke (IS), monitoring them from hospital admission to three months post-discharge. The primary aim was to evaluate swallowing recovery, clinical outcomes, and mortality risk in severe post-IS dysphagia over three months.

**Methods:**

Retrospective cohort study of 48 IS patients admitted to the Emergency Room (ER) of a tertiary hospital with persistent dysphagia (Functional Oral Intake Scale [FOIS] 1–4 at three weeks). Data included FOIS/EAT-10 scores, feeding methods, complications, and survival. Outcomes were analyzed at admission, three weeks, and three months.

**Results:**

Mortality: 50% died by 3-months, associated with older age, high NIHSS scores (median 16), and pre-existing neurological deficits (p < 0.05). 62.5% of survivors resumed oral intake (FOIS ≥ 5), but 75% reported persistent dysphagia (EAT-10 ≥ 3). 25% developed pneumonia; 33% received gastrostomy despite guidelines. Vertebrobasilar strokes showed lower mortality (p = 0.019).

**Conclusion:**

Severe post-IS dysphagia carries high mortality and prolonged functional impairment. While partial recovery occurs, dependence on alternative feeding and complications persist, underscoring the need for multidisciplinary follow-up.

## Highlights

• High Mortality Risk: Half of patients with severe dysphagia died within 3-months, linked to age, NIHSS scores, and prior neurological deficits.
• Recovery Patterns: Partial swallowing improvement occurred in 62.5% of survivors, yet 75% had persistent dysphagia symptoms.
• Clinical Gaps: Only 33% received guideline-recommended gastrostomy, and 25% developed aspiration pneumonia.
• Telehealth Utility: EAT-10 via telehealth captured dysphagia symptoms but underscores the need for adjunctive instrumental assessments.
• Prognostic Insights: Vertebrobasilar strokes had lower mortality despite severe deficits, suggesting infarct volume may modulate outcomes.

## Introduction

Stroke is a critical medical condition that remains a leading cause of mortality and long-term disability worldwide. Each year, approximately 13.7 million strokes occur globally, making stroke the second most common cause of death, responsible for 5.7 million fatalities^1^^,^^2^. In Brazil, between January and November 2024, around 177,590 individuals were hospitalized due to stroke, with 25,207 recorded deaths, reinforcing its position as the primary cause of mortality and disability in the country^2^^,^^3^. Among stroke subtypes, Ischemic Stroke (IS) is the most prevalent, accounting for 85 % of cases. It occurs when a thrombus or embolus obstructs a cerebral blood vessel, leading to ischemia, oxygen and glucose deprivation, and subsequent neuronal cell death^4^.

Multiple risk factors contribute to stroke incidence, including hypertension, overweight, sedentary lifestyle, diabetes mellitus, smoking, excessive alcohol consumption, advanced age, and male sex^5^. Clinically, stroke commonly manifests as unilateral weakness or tingling in the face, arm, or leg, speech disturbances, mental confusion, balance impairment, and sudden, severe headache^4^^,^^5^. Given the potential for irreversible neurological deficits, patient assessment is crucial. The National Institutes of Health Stroke Scale (NIHSS) is widely used to quantify neurological impairment, with scores ranging from 0 (no deficit) to 42 (severe impairment), assisting clinicians in evaluating patient status and prognosis^6^.

Among the disabling sequelae of stroke, neurogenic oropharyngeal dysphagia is particularly concerning. It has been reported in up to 90 % of cases and affects approximately 50 % of patients in the acute post-stroke phase^7^, ^8^, ^9^. Dysphagia results from impaired coordination of motor and sensory functions essential for safe swallowing, increasing the risk of malnutrition, dehydration, and pulmonary complications. Several factors have been associated with post-stroke dysphagia, with literature highlighting pre-existing neurological impairments, advanced age (> 72-years), NIHSS score > 14, stroke location and extent, dysarthria, and facial paralysis as major predictors of persistent swallowing dysfunction^7^.

The inability to swallow safely necessitates modifications in diet consistency and, in severe cases, the implementation of an Alternative Feeding Method (AFM)^1^. Post-stroke malnutrition is a significant concern, as it is strongly correlated with higher mortality rates and poor clinical outcomes^10^, ^11^, ^12^. Enteral Nutrition (EN) is recommended to ensure adequate caloric and nutritional intake for patients unable to maintain oral feeding^10^. While EN does not prevent aspiration, it does not increase the incidence of aspiration pneumonia^11^.

The American Heart Association/American Stroke Association (AHA/ASA) Guidelines for Stroke Rehabilitation and Recovery in Adults recommend Nasogastric Tube Feeding (NGT) for patients with dysphagia persisting beyond seven days^13^. If dysphagia extends beyond three weeks, Gastrostomy (GTT) is suggested to reduce the risk of aspiration pneumonia, in-hospital complications, and adverse outcomes^9^^,^^14^^,^^15^.

Studies reveal that approximately two-thirds of stroke patients with severe dysphagia fail to recover swallowing function within seven days, necessitating Enteral Nutrition (EN) for nutritional support^16^. Additionally, about one-third of these patients do not regain swallowing ability within 30-days, requiring long-term alternative feeding methods. One month post-stroke, two-thirds of dysphagic patients still require dietary modifications, and very few recover their pre-stroke diet^11^^,^^16^^,^^17^.

Key predictors of persistent severe dysphagia include advanced age, dysarthria, NIHSS score at admission, stroke location ‒ particularly lesions in the frontal operculum and insular cortex ‒ initial aspiration risk, and early impairment of oral intake^9^^,^^16^. Long-term follow-up of these patients may enable better prediction of swallowing outcomes and survival, offering critical insights for medical and speech-language pathology decision-making.

Thus, this study aims to evaluate the progression of swallowing function and clinical outcomes in ischemic stroke patients with severe dysphagia, following them from hospital admission to three months post-discharge. The goal is to determine swallowing prognosis and the relationship between dysphagia and survival in this population.

## Material and methods

This is a retrospective longitudinal clinical study. Data were collected from patients admitted to the Emergency Room (ER) of the Central Institute of the Hospital das Clínicas, School of Medicine, University of São Paulo (ICHC-FMUSP), Brazil, with a medical diagnosis of Acute Ischemic Stroke (AIS) and severe oral intake restriction, who were attended by the Division of Oral Motor Disorders of the same Institution. The study was approved by the Ethics Committee for the Analysis of Research Projects of the Institution (CAPPesq HCFMUSP – Process n° 3691,262). As this study was based on medical record analysis, the requirement for a written Informed Consent Form (ICF) from participants was waived.

### Participants

The study included patients admitted to the ER of a high-complexity hospital between May 2023 and May 2024, with a diagnosis of ischemic stroke and a maximum of 48 h since the onset of the stroke. The inclusion criteria were as follows: a) Confirmed diagnosis of ischemic stroke by imaging and clinical examination; b) Presence of dysphagia with severe oral intake restriction at the time of the assessment and three weeks after; c) Clinical stability, as per the medical records; d) Age over 18 years. The exclusion criteria were: a) Late hospital admission (> 48 h after stroke onset); b) Hemorrhagic stroke or recurrent stroke during the study period; c) Presence of functional swallowing or recovery of swallowing after three weeks; d) Functional or normal swallowing at the assessment; e) Need for endotracheal intubation prior to swallowing assessment; f) Surgical procedures involving the head and neck region; g) Pre-existing oropharyngeal dysphagia.

In accordance with the established protocols of the institution where the study was conducted, all patients admitted with ischemic stroke underwent a dysphagia screening process. This process was designed to identify those at risk of swallowing difficulties and ensure timely intervention. Specifically, patients who met any of the following criteria ‒age ≥ 69-years, NIHSS ≥ 9, Item 1. A score on the NIHSS ≥ 2, a history of previous neurological disease, or suspected Wallenberg syndrome patients were kept fasting while awaiting swallowing assessment. For patients who did not meet these criteria, the Water Glass Test (100 mL)^18^ was performed to assess swallowing function.

### Data collection

For the development of this study, all patients who met the inclusion criteria underwent assessment, which involved the collection of clinical and swallowing parameters. The following clinical variables were considered in the analysis: sex, age, severity of Ischemic Stroke (IS) as determined by the National Institutes of Health Stroke Scale (NIHSS)^19^, presence of prior neurological impairment (related to dementia and/or previous stroke), the affected area of the stroke’s blood supply (carotid or vertebrobasilar territory), stroke laterality, presence/absence of hemorrhagic transformation, and procedures performed (thrombolysis and/or thrombectomy). All stroke-related data were confirmed by a neurologist with extensive professional experience in the field.

The NIHSS (National Institutes of Health Stroke Scale)[6] is commonly used by healthcare professionals to assess the severity of a stroke and to monitor the patient's recovery over time. It is a simple tool that can be consistently applied at the bedside by the entire multidisciplinary team. This scale is a systematically used instrument that allows for a quantitative evaluation of the neurological deficits related to a stroke. The scale comprises 11-items that assess various abilities, including level of consciousness, eye movement, visual field, facial paralysis, motor function of the arms and legs, ataxia, sensation, language, speech, and extinction (neglect). Each item is scored on a scale ranging from 0 to 2, 0 to 3, or 0 to 4, depending on the ability being assessed. A score of 0 indicates normal function, while a higher score indicates greater neurological dysfunction. The scores for each ability are summed at the end to obtain a total value, which can range from 0 to 42 points. A score of 0 indicates no evidence of neurological deficit, while a score of 42 indicates that the patient is in a coma and unresponsive. It is important to note that the NIHSS does not include an evaluation of swallowing function.

### Clinical swallowing assessment

The clinical evaluation of swallowing was performed by Speech-Language Pathologists (SLPs) of the Division of Oral Myology of the same Institution within 48 h of hospital admission. All participating SLPs have extensive experience in the field and have successfully completed specific training tests. The clinical swallowing evaluation at the institution is conducted according to the Dysphagia Risk Evaluation Protocol, Screening Version (DREPs)^20^. DREPs is a Brazilian protocol, published and validated in 2019, designed for the early assessment of the risk of penetration/aspiration at the bedside. Its application involves offering controlled volumes of water and puree to assess the patient's ability to swallow safely.

The Functional Oral Intake Scale (FOIS)^21^ was used to determine the functional level of swallowing. FOIS is a simple-to-apply, highly recognized, and validated instrument since 2005, specifically designed for use in cases of dysphagia following stroke. The FOIS grades the functionality of oral feeding across levels, with scores ranging from 1 to 7. A score of 1 indicates the inability to consume food orally, while a score of 7 indicates oral feeding without restrictions.

All patients who met the clinical criteria for a swallowing evaluation were assessed within 48 h of the ischemic stroke onset (ictus) and again after 3-weeks from the initial assessment. This study only included patients who presented with severe oral intake restrictions at the initial evaluation (FOIS scores 1 to 4)^21^ and who continued to have significant dietary restrictions (FOIS scores 1 to 4)^21^ after 3-weeks. Additional variables collected during the initial assessment included: presence/absence of dysphonia, altered gag reflex, weak cough reflex, vocal alterations after swallowing water (5, 10, or 20 mL), dysarthria, aphasia, facial paralysis, and reduced level of consciousness during the initial speech-language evaluation.

The three-week cutoff was established based on the Stroke Association Guidelines, which indicate that patients with severe dysphagia persisting for this duration may require permanent alternative feeding methods, such as gastrostomy^13^. According to these guidelines, unresolved severe dysphagia after three weeks is associated with an increased risk of complications. Therefore, this study included only patients who exhibited severe oral intake restriction and, after the three-week period, required a long-term alternative feeding method. This approach enabled a more accurate analysis of clinical outcomes.

During the inpatient follow-up, the following process indicators were collected: type of feeding method at the time of evaluation and after, performance of Gastrostomy (GTT) during hospitalization, number of days from the swallowing evaluation until resumption of oral intake, and FOIS scores at the time of swallowing evaluation and after three weeks. The clinical outcome data collected included: hospital discharge, hospital transfer, and death during hospitalization. At the time of discharge or transfer, all patients underwent a final swallowing re-evaluation prior to discharge.

### Patient follow-up

All participants were followed by the Division of Oral Myology for three weeks after the initial evaluation and underwent a re-evaluation 90-days after the speech-language assessment. During this follow-up, clinical and swallowing data were collected using a standardized questionnaire, and the Eating Assessment Tool (EAT-10) was administered via telehealth. In all cases, the patient was accompanied by their primary caregiver, and a standardized administration protocol was followed. The EAT-10 was completed by the patient whenever feasible, with the caregiver assisting as needed ‒ particularly in cases involving cognitive, linguistic, or physical impairments ‒ to ensure accurate and reliable responses. This consistent method allowed for the uniform collection of patient-reported outcomes while reflecting real-world clinical conditions. The standardized questionnaire included questions related to general health status, need for re-hospitalization, nutritional status, swallowing ability, feeding method, and follow-up with other healthcare professionals.

The Eating Assessment Tool (EAT-10)^22^ is a widely used self-assessment questionnaire designed to evaluate swallowing difficulties and the risk of dysphagia. It was developed as a quick and simple screening tool to identify swallowing-related issues across various health conditions. The EAT-10 has been validated in several studies for different populations, including individuals with neurological conditions such as stroke. It also demonstrates high reliability and sensitivity for detecting dysphagia. The questionnaire consists of 10 items, with scores ranging from 0 (no problem) to 4 (severe problem), resulting in a maximum score of 40. Higher scores indicate greater swallowing difficulties. A total score of 3 or higher suggests the presence of dysphagia.

### Data analysis

The collected data were analyzed using SPSS software, version 29. Quantitative data were subjected to descriptive analysis (mean, standard deviation, median, and interquartile range for continuous data; total count and percentage for categorical data). Given the non-normal distribution of the data and the sample size, non-parametric tests were employed for inferential analysis, which included: 1) Comparisons between patients classified as FOIS levels 1 and 2 versus FOIS levels 3 and 4 at the post-stroke evaluation, using the Mann-Whitney *U* test for continuous variables and Pearson's Chi-Square test for categorical variables; 2) Comparisons of results obtained at each of the three evaluation time points (Initial Assessment, 3-weeks, and 3-months) using repeated measures ANOVA, with post-hoc pairwise analysis and Bonferroni correction applied when significant differences were found; and 3) Comparisons between patients who progressed to death and those who did not, using the Mann-Whitney *U* test for continuous variables and Pearson's Chi-Square test for categorical variables. Given the relatively small sample size of deceased patients, a multivariate regression model was not performed to analyze mortality risk, as such models typically require a larger number of events to provide reliable estimates when adjusting for multiple confounding variables.

Missing data for deceased patients at the follow-up evaluations (FOIS and EAT-10 scores) were handled using multiple imputation, performed automatically in SPSS. This method generates multiple datasets with imputed values based on the observed data, and the imputed data were used in all subsequent analyses.

Survival analysis was performed using the Kaplan-Meier method to assess differences in survival rates between patients stratified by NIHSS levels at the post-stroke evaluation. The variable time_to_event was created to represent the time until death or the last follow-up, where time = 1 corresponds to the 3-week follow-up, and time = 2 corresponds to the 3-month follow-up. The event of interest was death, with patients who did not die being censored at their last available follow-up time. The variable status_death indicated whether a patient had died at any follow-up time (1 = deceased, 0 = censored). Kaplan-Meier survival curves were generated for each NIHSS stratum, and differences in survival between groups were assessed using the log-rank test.

The significance level for all analyses was set at 5 %.

## Results

**Fig. 1:**
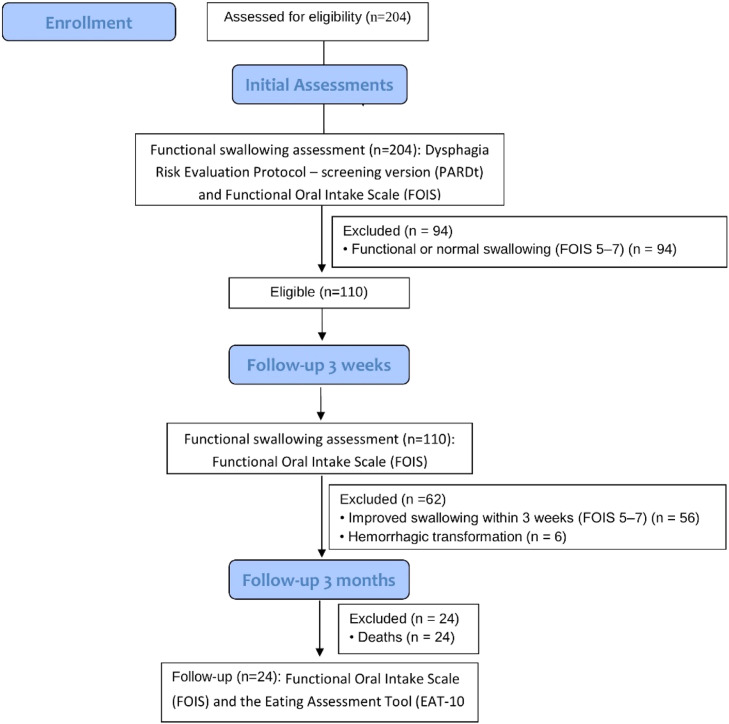
Flow diagram of patient inclusion and exclusion with corresponding numbers.

Between January and August 2024, a total of 204 evaluations were conducted for patients diagnosed with Ischemic Stroke (IS). Among these, 94 patients (46.07 %) presented with either mild dysphagia, functional swallowing, or normal swallowing on initial assessment, corresponding to FOIS levels 5, 6, or 7. The remaining 110 patients (53.93 %) were diagnosed with dysphagia, classified as FOIS levels 1 to 4 at the time of evaluation. Of the dysphagic group, 56 patients (50.90 % of the dysphagic subgroup, or 27.45 % of the total sample) demonstrated recovery of swallowing function within three weeks following the initial assessment. The other 54 patients (49.10 % of the dysphagic subgroup, or 26.47 % of the total sample) continued to present with dysphagia (FOIS levels 1 to 4) at both the initial and the three-week follow-up assessments. Among these 54 patients, six experienced hemorrhagic transformation of the ischemic stroke and were therefore excluded from the study. The final sample comprised 48 patients with persistent dysphagia three weeks post-ischemic stroke (Fig. 1).

The final sample consisted of 48 patients with persistent dysphagia three weeks after an ischemic stroke. For analytical purposes, participants were stratified based on their performance in the post-stroke initial swallowing assessment, specifically according to the Functional Oral Intake Scale (FOIS): individuals classified as FOIS levels 1 and 2 were grouped together, as were those classified as FOIS levels 3 and 4. Descriptive and comparative analyses of clinical and demographic variables were conducted for the total sample and between these two subgroups, as detailed in Table 1. The cohort was predominantly elderly, with a tendency toward left-sided carotid circulation involvement. A notable proportion of patients had pre-existing neurological conditions, suggesting a more complex clinical profile. High NIHSS scores were frequent, indicating moderate to severe stroke severity. Dysarthria and facial paralysis were the most common neurological deficits, underscoring the substantial functional impact of the stroke on communication and orofacial motor control.

**Table 1:** Sample characterization at the post-stroke initial swallowing assessment.

**Demographic and clinical data**	**Total sample (*n* = 48)**	**FOIS levels 1 and 2 (*n* = 35)**	**FOIS levels 3 and 4 (*n* = 13)**	**p-value**^a^
**Sex**				
Male	24 (50 %)	15 (42.86 %)	9 (69.23 %)	0.104
Female	24 (50 %)	20 (57.14 %)	4 (30.77 %)	
**Age**				
Median (Q1‒Q3)	72.5 (66.75‒81.5)	72 (67.5‒81.75)	73 (52‒77)	0.260
**Age categorization**				
≤ 70 years	20 (41.67 %)	14 (40 %)	6 (46.15 %)	0.701
≥ 71 years	28 (58.33 %)	21 (60 %)	7 (53.85 %)	
**OTI (after assessment)**	12 (25 %)	9 (25.71 %)	3 (23.08 %)	0.851
**Location and lateralization of stroke**				
Anterior carotid circulation ‒ right	14 (29.2 %)	12 (34.28 %)	2 (15.38 %)	0.227
Anterior carotid circulation ‒ left	20 (41.7 %)	15 (42.85 %)	5 (38.46 %)	
Posterior vertebrobasilar circulation	14 (29.2 %)	8 (22.87 %)	6 (46.2 %)	
**Previous neurologic deficit**	20 (41.67 %)	14 (40 %)	6 (46.15 %)	0.701
**NIHSS score**				
Median (Q1‒Q3)	16 (11.75–20)	17 (13–20.5)	13 (9–18)	0.160
**NIHSS categorization**				
0‒5	2 (4.16 %)	1 (2.86 %)	1 (7.7 %)	0.070
6‒13	15 (31.25 %)	8 (22.86 %)	7 (53.84 %)	
> 14	31 (65.58 %)	26 (74.28 %)	5 (38.46 %)	
**Dysarthria**	45 (93.75 %)	32 (91.43 %)	13 (100 %)	0.276
**Facial palsy**	43 (89.58 %)	31 (88.57 %)	12 (92.31 %)	0.706
**Aphasia**	27 (56.25 %)	21 (60 %)	6 (46.15 %)	0.390
**Thrombolysis**	16 (33.33 %)	11 (91.43 %)	5 (38.46 %)	0.646
**Thrombectomy**	8 (16.67 %)	6 (17.14 %)	2 (15.38 %)	0.885

n, Number of participants; %, Percentage; OTI, Orotracheal Intubation.

a Mann-Whitney *U* test.

**Fig. 2:**
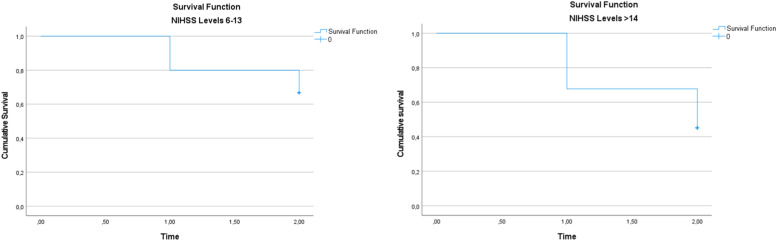
Kaplan-Meier survival curves for patients stratified by NIHSS levels (NIHSS 6–13 vs. ≥ 14).

All participants included in the study were followed by the Speech-Language Pathology team until hospital discharge. Each patient remained hospitalized for a minimum of three weeks, enabling continuous speech-language monitoring, which comprised swallowing rehabilitation sessions, periodic reassessments, and clinical support in decision-making ‒ particularly regarding the need for prolonged alternative feeding methods due to persistent dysphagia (FOIS scores ranging from 1 to 4). During the hospital stay, 16 patients (33.33 %) died, 2 patients (4.17 %) were discharged home, and 30 patients (62.5 %) were transferred to other facilities to continue rehabilitation. Consequently, a total of 32 patients remained in the study and proceeded to the next phase, which included a follow-up assessment three months after the onset of ischemic stroke. At the three-month follow-up, 8 additional patients had died, while 24 underwent clinical reassessment. By the end of the study, the total number of deaths reached 24 patients, representing 50 % of the initial sample. Of these 24 patients, 17 had been classified as FOIS levels 1 and 2 at the post-stroke evaluation. The survival curves for patients stratified by NIHSS levels are shown in Fig. 2. The Kaplan-Meier analysis demonstrates differences in survival rates between the groups, with statistical significance assessed by the log-rank test.

Table 2 presents the progression of swallowing functionality across the three time points of swallowing evaluation: the initial assessment following ischemic stroke onset, the reassessment after three weeks, and the final evaluation after three months. At the three-month follow-up, half of the initial sample had died, highlighting the severity of clinical outcomes in this population. Among the surviving patients, clinical reassessment revealed that although dysphagia persisted in the majority of cases, a gradual functional improvement in swallowing was observed across the three assessment points. Notably, an increasing proportion of patients became able to resume some level of oral intake over time, reducing the dependency on alternative feeding methods. Despite the high initial prevalence of severe dysphagia requiring exclusive alternative feeding, many patients progressed to safer and more functional oral intake. Statistical analysis confirmed a significant improvement in swallowing function over time, particularly between the initial evaluation and the three-month follow-up (*p* = 0.002), underscoring the potential for recovery even in cases of initially severe impairment.

**Table 2:** Functional level of swallowing.

**FOIS Level**	**Initial Assessment, n (%)**	**3-weeks, n (%)**	**3-months n (%)**	**p-value**	**Pairwise Comparison**
1	8 (16.67)	9 (25.71)	3 (12.5)	**<0.001**^a^	Initial vs. 3-weeks *p* = 0.967
2	27 (56.25)	12 (34.29)	2 (8.33)		
3	4 (8.33)	7 (20)	4 (16.67)		Initial vs. 3-months ***p* = 0.002**^b^
4	9 (18.75)	7 (20)	2 (8.33)		
5	‒	‒	9 (37.5)		3-weeks vs. 3-months ***p* = 0.040**^b^
6	‒	‒	2 (8.33)		
7	‒	‒	2 (8.33)		

FOIS, Functional Oral Intake Scale; n, number of participants.

a Significant difference ANOVA test.

b Significant difference Pairwise post-hoc test Bonferroni correction.

The results presented in Table 3 refer to the three-month follow-up after the initial assessment. Regarding medical complications, among the 24 patients followed longitudinally, 25 % developed pneumonia after hospital discharge, and 25 % required rehospitalization. In terms of nutritional status, 50 % of the patients reported weight loss. Importantly, among those, 25 % had a FOIS score of 5 or 6, suggesting a significant nutritional compromise likely associated with dysphagia. Furthermore, 75 % of the patients scored 3 or higher on the EAT-10 questionnaire, indicating the persistence of swallowing difficulties three months post-stroke. The most frequently endorsed items included “I get food stuck in my throat” (58.33 %) and “I cough when I eat” (79.16 %). Finally, 50 % of the patients remained under swallowing follow-up at three months, while 33.3 % of those receiving alternative feeding (e.g., enteral nutrition) were not engaged in swallowing rehabilitation therapy.

**Table 3:** Follow-up results (3-months).

**Eating Assessment Tool**	**Total sample ‒ survivors (*n* = 24)**	**FOIS levels 1 and 2 at initial assessment (*n* = 18)**	**FOIS levels 3 and 4 at initial assessment (*n* = 6)**	**p-value**
Pneumonia	6 (25 %)	5 (14.3 %)	1 (16.7 %)	0.586
Rehospitalization	6 (25 %)	3 (8.6 %)	5 (88.3 %)	0.102
Weight				
Gain	9 (37.5 %)	7 (38.9 %)	2 (33.3 %)	0.929
Loss	12 (50 %)	9 (50 %)	3 (50 %)	
Equal	3 (12.5 %)	2 (11.1 %)	1 (16.7 %)	
Feeding method				
Oral	15 (62.5 %)	12 (66.7 %)	3 (50 %)	0.449
Mixed	4 (16.7 %)	2 (11.1 %)	2 (33.3 %)	
Exclusive alternative method	5 (20.8 %)	4 (22.2 %)	1 (16.7 %)	
EAT 10 score > 3	18 (75 %)	13 (72.2 %)	5 (88.3 %)	0.586
Ongoing swallowing rehabilitation	12 (50 %)	9 (50.0 %)	3 (50.0 %)	>0.999

EAT 10, Eating Assessment Tool; n, number of participants.

Table 4 presents a comparative analysis between patients who died and those who survived, based on data collected during the initial clinical assessment. The gender distribution revealed a higher prevalence of males among patients who died (54.17 %) compared to those who survived (45.83 %). In addition, Orotracheal Intubation (OTI) following the assessment was more frequent in the deceased group (29.17 %) than in the surviving group (20.83 %). Stroke location also proved to be a relevant factor. Carotid circulation was more commonly affected in the deceased group (79.17 %) compared to the non-deceased group (62.5 %). Moreover, the presence of previous neurological conditions emerged as an important risk factor: patients with a neurological history had a higher mortality rate (58.33 %) than those who survived (25 %). Regarding stroke severity measured by the NIHSS, patients who died had higher scores (mean = 17.304; median = 16.5) compared to survivors (mean = 13.875; median = 15.5), with 70.23 % scoring 14 or above. Overall, the data demonstrated that factors such as age, stroke location, NIHSS severity, history of neurological conditions, and the need for intubation are associated with increased risk of mortality. These findings provide greater insight into the factors influencing the prognosis of patients with severe dysphagia following stroke.

**Table 4:** Comparison of survivors and non-survivors.

	**Survivors (*n* = 24)**	**Non-Survivors (*n* = 24)**	**p-value**
**Sex**			
Male, n ( %)	11 (45.83)	13 (54.17)	0.564
Female, n ( %)	13 (54.17)	11 (45.83)	
**Age**			
Median (Q1‒Q3)	72 (62–78)	79 (67–88)	***0.050***^a^
**OTI (after assessment)**	5 (20.83)	7 (29.17)	0.505
**Location of stroke**			
Carotid circulation	15 (62.5)	19 (79.17)	0.119
Vertebrobasilar circulation	9 (37.5)	5 (20.83)	
**Previous neurologic deficit**	6 (25.0)	14 (58.33)	***0.019***^b^
**NIHSS**			
Median (Q1‒Q3)	15.5 (8‒19)	16.5 (13‒21)	0.066
**NIHSS categorization**			
0‒5	2(8.33)	0	0.308
6‒13	8(33.33)	7(29.17)	
> 14	14(58.33)	17(70.23)	
**Dysarthria**	22(91.67)	23(95.83)	0.551
**Facial palsy**	21(87.50)	22(91.67)	0.637
**Aphasia**	14(58.33)	13(54.16)	0.771
**Thrombolysis**	5(20.83)	11(45.84)	0.066
**Thrombectomy**	6(25.0)	2(8.33)	0.121
**FOIS (first assessment)**			
4	2(8.33)	7(29.17)	0.063
3	4(16.67)	3(12.5)	
2	13(54.17)	14(58.33)	
1	5(20.83)	3(12.5)	

n, number of participants; %, Percentage; OTI, Orotracheal Intubation.

a Statistically significant difference between groups according to the Mann-Whitney *U* test for independent samples.

b Statistically significant difference between groups according to Pearson’s Chi-Square test.

## Discussion

Dysphagia is a significant complication among elderly patients who have experienced an Ischemic Stroke (IS), with prevalence rates ranging from 42 % to 67 % in this population. It increases the risk of pulmonary complications, predisposition to additional comorbidities, prolonged hospitalization, and mortality^23^. Older adults are particularly vulnerable to dysphagia due to age-related neurological changes. Aging is associated with cerebral atrophy, deterioration of neural functions, and loss of muscle mass ‒ factors that compromise the swallowing process by affecting both the oral and pharyngeal phases^23^^,^^24^. The present findings revealed that 62.50 % of patients had high scores on the NIH Stroke Scale (NIHSS), indicating severe neurological deficits and substantial functional impairment. The association between elevated NIHSS scores and severe dysphagia has been documented in previous studies, which suggest that greater neurological impairment serves as a predictor of poorer swallowing recovery outcomes^9^^,^^25^^,^^26^.

Regarding stroke location, a predominance of lesions in the carotid circulation and a higher frequency of left-sided lesions were observed. Studies have shown that strokes affecting the anterior circulation ‒ supplied by the carotid arteries ‒ particularly on the left side, are more likely to impact brain regions critical for the neural control of swallowing, such as the insular cortex, frontal operculum, and basal ganglia. Damage to these areas exacerbates swallowing dysfunction^27^. Furthermore, carotid circulation strokes are often associated with more extensive and potentially devastating lesions due to the role of this vascular territory in supplying critical brain regions^27^^,^^28^.

In contrast, vertebrobasilar circulation strokes ‒ more commonly observed among survivors in this study ‒ are typically linked to severe neurological deficits^29^, although they demonstrated a lower impact on early mortality in this cohort. This may be partly explained by the typically smaller infarct volumes and more localized brainstem involvement in vertebrobasilar strokes, as previously described in other studies^30^^,^^31^, which reported lower early mortality rates in the absence of complete occlusion or hemodynamic instability. Recent evidence also highlights the importance of the integrity of the unaffected hemisphere ‒ particularly the contralateral frontal operculum ‒ in predicting swallowing recovery^27^. These associations have important clinical implications, as lesion location within specific brain structures may inform both prognosis and the development of targeted rehabilitation strategies.

The presence of dysarthria and facial paralysis among post-stroke dysphagic patients is consistent with findings from recent studies. One study, in particular, demonstrated that dysarthria was independently associated with the persistence of severe oral intake restrictions in patients with acute stroke^9^. However, in the present cohort, the isolated presence of dysarthria was not sufficient to distinguish between mortality and survival outcomes. This relationship may be explained by the shared involvement of muscle groups responsible for speech motor production, facial expression, and the physiological mechanisms of swallowing^9^^,^^32^.

The interplay between dysarthria and dysphagia in the context of ischemic stroke is complex and closely related to neurological impairments affecting motor pathways that control both speech and swallowing musculature^33^. Stroke can lead to damage in critical areas of the central nervous system involved in motor planning and neuromuscular coordination, including the motor cortex, brainstem, and cranial nerves^34^. Dysarthria, characterized by impaired speech articulation, typically results from muscle weakness or lack of motor control ‒ factors that similarly affect the musculature involved in swallowing, thereby contributing to dysphagia. This comorbidity significantly increases the risk of aspiration, malnutrition, and aspiration pneumonia^31^^,^^35^.

The three-week follow-up revealed that approximately half of patients with post-stroke dysphagia experienced partial recovery of swallowing function, suggesting that a subset of individuals with severe dysphagia following ischemic stroke has potential for short-term functional improvement, albeit with limitations. This progression aligns with findings from previous studies indicating that early recovery of swallowing may result from functional compensation and neuroplasticity in adjacent brain regions, particularly during the subacute phase post-stroke, as neural networks disrupted by the infarct begin to re-establish functional connectivity^36^^,^^37^.

By the end of the three-month follow-up period, almost two-thirds of the remaining patients were able to resume oral feeding, albeit with dietary consistency modifications. However, EAT-10 scores revealed that 75 % of these individuals still perceived a risk of dysphagia, and 25 % had experienced aspiration pneumonia. These findings indicate that, despite the persistence of dysphagic symptoms in a considerable portion of patients, there is measurable improvement in swallowing capacity over time. This underscores the importance of continuous speech-language pathology intervention. Early identification of dysphagia and timely therapeutic stimulation may promote the reconnection of neural networks in adjacent areas, enabling partial or even complete functional compensation for damaged regions^36^^,^^37^.

Given the retrospective nature of this study and limitations in access to instrumental evaluations, follow-up assessment ‒ i.e., EAT-10 ‒ was conducted via telehealth, with the presence of caregivers to support consistent administration. Although clinicians may express uncertainty about the absence of instrumental methods, especially for detecting silent aspiration, previous studies have demonstrated the clinical reliability of remote dysphagia screening tools. Studies point out that telehealth evaluations of post-stroke dysphagia closely matched in-person assessments^38^, while others confirm the feasibility and clinical value of telepractice in dysphagia management, particularly under resource-constrained conditions^39^. These findings support the validity of the methodological approach and reflect an increasingly accepted practice in dysphagia care.

The prolonged need for alternative nutritional support observed in this cohort reflects the severity of dysphagia frequently seen in the post-stroke population. According to the American Stroke Association, when oral intake remains significantly limited beyond the initial weeks, the initiation of long-term enteral nutrition via gastrostomy is recommended^13^. Although gastrostomy does not differ significantly from nasoenteric feeding in terms of mortality or dependency^9^^,^^14^, it has been associated with reduced treatment failure rates^9^^,^^14^, increased feeding safety^40^, improved nutritional delivery^41^, and shorter hospital stays^13^^,^^14^. Despite these advantages, only a minority of patients in the present study received a gastrostomy, reflecting the absence of a standardized institutional protocol to guide timely decision-making. In this setting, while speech-language pathologists routinely assess and recommend enteral feeding strategies, the decision to proceed with gastrostomy is made by the attending physician, often without a clear timeline or multidisciplinary consensus. This gap may contribute to delayed or inconsistent management.

Despite clear clinical indications, many patients in this study did not receive long-term enteral feeding and instead resumed oral intake without supervision by a speech-language pathologist. Recent prognostic models, such as the Predictive Swallowing Score (PRESS)^16^, offer valuable guidance for these clinical decisions. The PRESS model uses a combination of factors ‒ including age, NIHSS score, lesion location (especially involving the frontal operculum), aspiration risk, and oral intake impairment ‒ to predict swallowing recovery and support early decisions about nasogastric or percutaneous endoscopic gastrostomy feeding. The model has demonstrated strong predictive accuracy and calibration, with almost two-thirds of patients with severe dysphagia showing no functional recovery by day 7, and one-third still impaired at day 30. Integrating models like PRESS into clinical practice may enhance decision-making, promote timely initiation of appropriate feeding strategies, and reduce complications arising from delayed or inappropriate transitions in nutritional support. This gap in care may have contributed to the incidence of aspiration pneumonia and unplanned readmissions, outcomes that are well-documented complications of severe dysphagia when airway protection is compromised^42^. Moreover, even among patients who advanced to less restrictive diets and perceived their general health as favorable, reports of weight loss were not uncommon. This suggests that functional improvements in oral intake may not always correspond to adequate nutritional status. Malnutrition, which is known to worsen prognosis and increase complication rates^10^^,^^11^, remains a significant concern in this population, with prevalence reported to exceed 48 % among post-stroke patients^37^.

These observations reinforce the need for structured multidisciplinary follow-up, including regular monitoring by speech-language pathologists and dietitians, to ensure safe dietary progression and prevent nutritional deficits. Early identification of dysphagia and implementation of targeted interventions can facilitate functional compensation, especially during the subacute phase of recovery^36^^,^^37^.

In this context, clinical decisions regarding the advancement of oral intake should not rely solely on the apparent return of swallowing function but must be guided by comprehensive assessments that integrate clinical, instrumental, and nutritional parameters. The mismatch between perceived functional recovery and actual swallowing safety or efficiency highlights a critical area where underestimation of dysphagia severity may lead to adverse outcomes, particularly in the absence of professional oversight. Furthermore, the data suggest that post-discharge management of dysphagia remains insufficiently structured. The transition from hospital to outpatient care often lacks continuity, with limited access to follow-up by specialized teams. This fragmentation of care may delay the detection of deteriorating nutritional status or silent aspiration, both of which can contribute to complications such as pneumonia and weight loss^42^^,^^43^.

Importantly, the findings support the notion that dysphagia in stroke survivors should be addressed not only as a transient complication but as a condition requiring long-term management strategies. While some individuals demonstrate spontaneous recovery due to compensatory activation of contralateral structures^36^^,^^37^, others may require prolonged rehabilitative efforts to achieve functional oral intake and reduce the risk of morbidity. Therefore, integrating speech-language pathology into the continuum of stroke care ‒ from acute hospitalization to rehabilitation and outpatient settings ‒ is essential. Similarly, ensuring timely nutritional interventions, including individualized dietary adaptations and appropriate decisions regarding enteral support, can play a crucial role in preventing deterioration of health status and promoting safe reintegration into daily life^9^^,^^14^^,^^40^^,^^42^^,^^44^.

This study has several limitations that should be considered when interpreting its findings. The relatively small sample size, drawn from a single healthcare institution, may limit the generalizability of the results to broader populations. Additionally, the retrospective design is inherently subject to information bias and potential gaps in clinical documentation. Reliance on existing medical records may have resulted in incomplete or inconsistent data, particularly regarding detailed lesion topography and nutritional biomarkers, which could have provided a more nuanced understanding of individual patient profiles.

A significant limitation is the absence of instrumental swallowing assessments, such as Videofluoroscopic Swallowing Study (VFSS) or Flexible Endoscopic Evaluation of Swallowing (FEES), which are generally considered superior to clinical bedside assessments due to their ability to provide dynamic, objective visualization of swallowing mechanics and better identify aspiration risk and pharyngeal dysfunction^44^. The lack of these instrumental evaluations reflects the real-world constraints of the clinical setting during the study period, where resource availability limited their routine use. Instead, the authors relied on validated clinical tools such as the Eating Assessment Tool-10 (EAT-10) and the Functional Oral Intake Scale (FOIS), which have demonstrated excellent internal consistency (Cronbach’s alpha = 0.96) and good test-retest reliability (Pearson correlation coefficients ranging from 0.72 to 0.91), supporting their use as reliable markers of swallowing function severity^45^^,^^46^. Nevertheless, the subjectivity inherent in these clinical tools may have reduced sensitivity in detecting subtle aspiration events or pharyngeal phase dysfunction compared to instrumental methods.

Moreover, although the three-month follow-up period provided valuable insights into early recovery patterns, it may not have captured longer-term functional trajectories or late-emerging complications. Variability in the timing and availability of speech-language pathology interventions also introduces potential confounding factors, as differences in access and intensity of therapy could have influenced individual outcomes. In addition, the exclusion of patients with hemorrhagic transformation ‒ due to their distinct pathophysiological and clinical characteristics ‒ may have introduced selection bias, potentially skewing the sample toward less severe ischemic stroke cases. While this methodological choice was intended to reduce heterogeneity and improve interpretability, it may limit the generalizability of the findings to more complex or mixed clinical presentations. Compounding these considerations, the high in-hospital mortality rate observed in this cohort (50 %) further constrains the applicability of results to broader stroke populations. This elevated mortality likely reflects the severe clinical profile of many patients included in the study ‒ such as those with significant comorbidities ‒ and may have biased the sample toward more acute or medically complex cases. As a result, the findings may be less representative of individuals with more favorable prognoses. Nonetheless, this high mortality rate highlights the importance of early identification and management of dysphagia as a critical prognostic factor and underscores the need for cautious interpretation when generalizing these outcomes to longer-term survivors or different clinical contexts.

Despite these limitations, the study offers several strengths that enhance the value and applicability of its findings. It was conducted within a real-world clinical context, thereby reflecting the constraints, decision-making processes, and variability inherent in routine care ‒ factors often absent in controlled clinical trials. This ecological validity increases the relevance of the results for everyday clinical practice. The use of validated clinical tools, such as the NIHSS and EAT-10, provided reliable markers for neurological and swallowing function and enabled consistent data collection across patients, supporting the robustness of functional assessments even in the absence of instrumental methods. Additionally, the longitudinal perspective ‒ with follow-up extending to three months ‒ allowed for the observation of recovery trends and the identification of delayed complications, offering a more comprehensive view of post-stroke swallowing outcomes beyond the acute phase. The inclusion of functional oral intake data also adds practical insight into patients' real-life eating abilities and their nutritional trajectories during recovery. Furthermore, the study focuses on a population that remains underrepresented in post-stroke dysphagia research ‒ patients with ischemic stroke in low-resource settings ‒ helping to fill a critical knowledge gap. These strengths not only reinforce the clinical relevance of the findings but also highlight the importance of integrating long-term, multidisciplinary care, including ongoing speech-language pathology support, into the management of dysphagic stroke survivors.

## Conclusion

This study highlights the high prevalence and clinical complexity of dysphagia in elderly patients following ischemic stroke, emphasizing its association with severe neurological impairment, specific lesion locations, and related complications such as aspiration pneumonia and malnutrition. Despite evidence of partial recovery over time, particularly within the first three months, persistent symptoms and adverse outcomes were common, underscoring the need for structured, multidisciplinary follow-up. The findings reinforce the importance of early identification and continuous management of dysphagia, advocating for integrated care strategies that involve speech-language pathologists and dietitians throughout the recovery continuum to optimize functional outcomes and prevent further health deterioration in this vulnerable population.

## Authors’ contributions

Gabriela Mourthé Félix: Responsible for data collection and analysis; interpretation of the results; writing the major portion of the paper.

Fernanda Chiarion Sassi: Responsible for supervising the research; interpretation of the results; writing major portion of the paper.

Carina Escudero: Contributed to data analysis and interpretation; responsible for revising the final version of the manuscript.

Karoline Kussik de Almeida Leite: Contributed to research experimental design and data interpretation; contributed to manuscript preparation.

Ana Paula Ritto: Contributed to data analysis and interpretation; organized the statistical analyses; contributed to manuscript preparation.

Cirley Novais Valente Junior: Contributed to data collection and analysis; contributed to manuscript revision.

Claudia Regina Furquim de Andrade: Responsible for the research and experimental design; contributed to data analysis and manuscript preparation.

## Funding

The authors confirm that this study did not receive any financial support or sponsorship. All data collection and analysis were conducted without external funding, and no institutional or third-party resources were used beyond routine clinical infrastructure.

## Data availability statement

The datasets generated and/or analyzed during the current study are available from the corresponding author upon reasonable request.

## Declaration of competing interest

The authors declare no conflicts of interest.
